# Patient satisfaction on tuberculosis treatment service and adherence to treatment in public health facilities of Sidama zone, South Ethiopia

**DOI:** 10.1186/1472-6963-13-110

**Published:** 2013-03-22

**Authors:** Zekariyas Sahile Nezenega, Yohannes H/Michael Gacho, Tadese Ejigu Tafere

**Affiliations:** 1 Department of health officer, Ambo University, P.O.BOX 19, Ambo, Ethiopia; 2 Department of health planning and health services management, Jimma University, P.O.Box 378, Jimma, Ethiopia; 3 BahirDar University, BahirDar, Ethiopia

**Keywords:** Tuberculosis, Satisfaction, Adherence

## Abstract

**Background:**

Patient compliance is a key factor in treatment success. Satisfied patients are more likely to utilize health services, comply with medical treatment, and continue with the health care providers. Yet, the national tuberculosis control program failed to address some of these aspects in order to achieve the national targets. Hence, this study attempted to investigate patient satisfaction and adherence to tuberculosis treatment in Sidama zone of south Ethiopia.

**Methods:**

A facility based cross sectional study was conducted using quantitative method of data collection from March to April 2011. A sample of 531 respondents on anti TB treatment from 11 health centers and 1 hospital were included in the study. The sample size to each facility was allocated using probability proportional to size allocation, and study participants for the interview were selected by systematic random sampling. A Pre tested, interviewer administered questionnaire was used to collect the data. Collected data was edited, coded and entered to Epi data version 3.1 and exported to SPSS version 16. Confirmatory factor analysis was done to identify factors that explain most of the variance observed in most of the manifested variables. Bivariate and Multivariate analysis were computed to analyze the data.

**Result:**

The study revealed 90% of the study participants were satisfied with TB treatment service. However, 26% of respondents had poor adherence to their TB treatment. Patient perceived on professional care, time spent with health care provider, accessibility, technical competency, convenience (cleanliness) and consultation and relational empathy were independent predictors of overall patient satisfaction (P < 0.05). In addition to this, perceived waiting time was significantly associated with patient satisfaction (Beta = 0.262). In multivariate analysis occupational status, area of residence, perceived time spent with health care provider, perceived accessibility, perceived waiting time, perceived professional care and over all patient satisfaction were significantly associated with adherence to TB treatment (P < 0.05). Moreover, patient waiting time at reception room (Adjusted OR = 1.022, 95% CI 1.009, 1.0035) and Patient treatment phase (Adjusted OR = 0.295, 95% CI 0.172, 0.507) were independent predictor of adherence to TB treatment.

**Conclusion:**

The finding of this study showed that patients’ perceptions on health care provider interaction had a significant influence on patient satisfaction and adherence to TB treatment. Moreover, absence of drugs and long waiting time had a negative outcome on patient adherence. Therefore, the problem needs an urgent attention from programme managers and health care providers to intervene the challenges.

## Background

Tuberculosis (TB) is a global health concern; nearly one-third of the global population is infected with Mycobacterium tuberculosis and at risk of developing the disease [1]. More than 90% of global TB cases and deaths occur in the developing world, where 75% of cases are in the most economically productive age group [2]. Ethiopia ranks seventh among the world’s 22 high-burden tuberculosis (TB) countries [3].

World Health Organization (WHO) recommends the Directly Observed Treatment Short-course (DOTS) strategy for control of TB. The strategy has been adopted by more than 180 countries and is considered as the most appropriate and cost-effective approach for TB control [4].

The WHO target for global TB control was to detect at least 70% of the estimated smear-positive TB cases and to achieve a treatment success rate of 85% in 2005. Only very few high TB burden countries have achieved both targets [5]. In Ethiopia it seems that the targets are missed. According to the Federal Ministry of Health annual report for 2010/2011, TB detection rate is 36.8% which is below the national target (59%) set for the year and TB success rate shows a downward fluctuation (from 84.0% to 82.5%) during the same period [6].

Patient compliance is a key factor in treatment success. In many countries, a significant proportion of patients stop treatment before completion, for various reasons. Promoting compliance through a patient-centered approach is much more effective than spending resources on defaulter tracing [7].

Studies have shown that, satisfied patients are more likely to utilize health services [8], comply with medical treatment [9], and continue with the health care providers [10]. Asking patients what they think about the care and treatment they have received is an important step towards improving the quality of care, and to ensuring that local health services are meeting patients’ needs [11]. Study conducted in Tigray region of northern Ethiopia shows that the poor quality of TB service delivery in public health facilities was key determinants of low adherence to treatment [12].

In the southern region of Ethiopia (Southern Nations Nationalities and Peoples’ Regional State), Even though, Directly Observed Treatment Short-course( DOTS) was introduced in 1996, evidence shows that one in five patients still continued to default from treatment [13]. Contrary to this the other study reported that between 1995 and 2004, the default and failure rates decreased from 26% to 6% and from 7% to 1%, respectively [14]. Nevertheless, to our knowledge there is no adequate information on patient satisfaction on TB treatment service and adherence to treatment in Ethiopia. Thus, this research was aimed at investigating the extent of patient satisfaction, treatment adherence and associated factors in a sample of tuberculosis treatment following patients in Sidama zone, South Ethiopia.

## Methods

### Study setting

A facility based cross sectional study was employed using quantitative method of data collection in Sidama zone of South nations, nationalities and peoples region from March to April 2011. The Southern Nations, Nationalities and Peoples’ Region has an area of 118,000 sq km. and 15.7 million people, constituting 20% of the nation’s total. It has 13 zones (province), 8 special woredas (district), 133 woredas (district), 22 town administrations and 3,851 rural villages [15].

### Sampling

The source population of the study was all TB patients on anti TB treatment in 11 public health centers and one hospital of Sidama zone. TB patients who were following their anti TB treatment for more than two or more weeks, and whose age is 15 years and above were included for study. The sample size was calculated using a single population proportion formula assuming, proportion of patient adherence to TB treatment is p (0.77), which give maximum sample size, from previous study conducted in Ethiopia [16]. Considering 5% margin of error (d) and confidence level of 95% (*z*^*a*^/2 = 1.96). Based on the above information a sample size was 272. However, since the target population was <10, 000 correction formula was applied which gives 253. Then sample size was multiplied by a factor of 2 to correct the design effect for the multi stage sampling and adding 10% non response rate, the final sample size was 556.

### Sampling technique

A multi stage stratified sampling technique was used with the strata of health facility exist in urban and rural. A total of 46 health centers and 2 hospitals were providing TB treatment service in the Zone during data collection period, of which 27 health centers were located in rural district and the remaining 19 health centre and the two hospitals in urban. Due to resource constraint 25% (12) of the health facilities (7 health centers from rural and 4 health centers and 1 hospital from urban) were selected randomly. The total sample size was allocated to the selected health centers and hospital based on the probability proportional to size of patients who started TB treatment service during two weeks priors to data collection. Then respondents were selected by systematic random sampling every 2 TB patients. The first respondent was the 2nd visitor of TB patient which selected by lottery method.

### Data collection

Trained 12 non health professionals and 3 B.Sc health professionals were used as data collectors and supervisors in this study.

### Measurements

For assessing adherence to medications a validated instrument developed by Morisky was used. Morisky Adherence Scale (MAS) is a 4-item self report scale with original binary response option (yes [1] and no [0]) and one open ended question was used for clarification of reason for non adherence. The classifications of the patients as Poor adherence, Patients compliant with fewer than four of the four items/MAS score 1–4, or Good adherence, Patients compliant with the four MAS item/MAS score 0, depended on the proportion of binary answers [17].

For assessing patient satisfaction a validated patient satisfaction questionnaire was adapted from Birhanu et al. 2010 and Grant N. Marshall & Ron D. Hays 1994 [18,19]. The measurement has 5 sub-scales: Consultation and Relational Empathy (10 items), Perceived technical competency and/ or professional care (11 items), Perceived Accessibility and convenience (9 items), Time spent with health care provider (2 items) and patient general satisfaction (5 items). Each item is measured by a five-point Likert scale. The questionnaire was translated into Amharic & Sidamagna and was retranslated into English to ensure its consistency.

The items of the scale were subjected to factor analysis by principle component analysis with varimax rotation to look into the underlying component. A factor loading of 0.4 as a cutoff point was used for principal component analysis to eliminate variables with low correlation from each factor. A reliability test was applied to examine the internal consistency of each factor separately. Based on Eigenvalues greater than one, eight components were identified as Consultation and Relational Empathy has 10 items with Cronbach’s alpha = 0.940, Perceived professional care has 8 items with Cronbach’s alpha = 0.898, Perceived technical competency has 2 items with Cronbach’s alpha = 0.892, perceived accessibility has 2 items with Cronbach’s alpha = 0.690), Perceived waiting time has 2 items with cronbah’s alpha = 0.738, Perceived convenience (cleanliness) has 2 items with Cronbach’s alpha = 0.686, Perceived time spent with health care provider has one item was used and Overall patient satisfaction with TB treatment service has 3 items with cronbah’s alpha =0.792.

#### Statistical analysis

The data was edited, coded, and entered using Epi data version 3.1 and exported to SPSS version 16. Using SPSS version 16, descriptive analysis (Mean ± SD, median and percentile for continuous variables and frequencies for categorical variables) was conducted. Confirmatory factor analysis was done to identify factors that explain most of the variance observed in a much of manifested variables. A factor loading of 0.4 was used as a cutoff point to eliminate variables with low correlation from each factor. Factors with eigenvalue less than one were discarded. Negatively worded questions were reverse scored (so that 1 = 5, 2 = 4, etc.). Thus high score always shows higher satisfaction. Analysis of regression (linear and binary logistic) was done to determine the predictors of patient satisfaction and treatment adherence. P-value less than 0.5 were used as cut off point.

#### Ethical consideration

The research was approved for scientific and ethical integrity by ethical review board of the college of public health and medical sciences of Jimma University. Participation in the study was voluntary and based respondent’s ability to give verbal informed consent.

## Result

### Socio-demographic characteristics

A total of 531 TB patients aged 15 years and older were responded to the interviewed, making a response rate of 95.5%. Two hundred ninety one (54.8%) of the respondents were male. The median age of the respondents was 28 years. Two hundred seventy two (51.2%) of the respondents were married, while 206 (38.8%) were single. Three hundred sixty six (68.9%) of the respondents were residing in rural area. More than a quarter 153(28.8%) of the respondents have attended up to second cycle education (grade 5–8); while 124 (23.1%) of them were illiterate. One hundred thirty five (25.4%) of the respondents were farmers and 127(23.9%) were house wife and 121 (22.8%) of them were students by occupation.

### General health service aspect and treatment phase

The mean distance of the health facilities from the respondent’s home was 2.67 kilometers with SD ±1.63 and the median travel time to reach health facilities on foot was 45 minute. About four in five (79.8%) of the respondents were traveled on foot to reach health facilities. Of the respondents 93.4% reached to the health facilities within 2 hours walk. The median waiting time of the respondents in reception room was 12 minute. The mean time spent for consultation with the health care provider was 7.12 minute with SD ±4.45. Of the respondents 376(70.8%) were on continuation phase whereas the remaining were on intensive phase at the time of interview. Among the total respondents 288 (54.2%) rate their current health status as very well, 234 (44.1%) as good and 9 (1.7%) as not good.

### Patient’s perceptions on the health care provider interaction

The mean score for perceived consultation and relational empathy was 35.88 with SD ±8.52. About 75% of the respondents were scored greater than 29 on perceived consultation and relational empathy scale for the 10 items of likert scale. Thus, almost 75% of the respondents were rated the perceived consultation and relational empathy from Good to Excellent. Similarly, the mean score for perceived professional care was 32.82 with SD ±5.20. Based on the percentile values 75% of the respondents were rated the perceived professional care from agree to strongly agree (Table 1).

**Table 1:** Patients perceptions on the health care provider interaction at public health facilities of Sidama zone, South Ethiopia, from March to April 2011, (N = 531)

**Subscale**	**No. items**	**25**^**th **^**percentile**	**50**^**th **^**percentile**	**75**^**th **^**percentile**	**Mean**	**SD**
**perceived consultation and relational empathy**	10	29	35	**43**	35.88	8.52
**perceived professional care**	8	32	33	**36**	32.82	5.20
**perceived technical competency**	2	6	8	**10**	7.66	2.44
**perceived accessibility**	2	7	8	**9**	7.68	1.86
**perceived waiting time**	2	6	8	**9**	7.16	2.37
**Perceived convenience**	2	8	8	**10**	8.46	1.39
**Perceived time spent with heath care provider**	1	4	4	**5**	3.88	1.02

### The overall Patient satisfaction

The overall patient satisfaction on TB treatment service was rated by five point likert scale from strongly disagree to strongly agree as shown in Table 2. The study showed that the mean score of the overall patient satisfaction on TB treatment service was 12.79 with SD ±2.30. The percentile values showed that 10% of the respondents were scored 10 or less for the satisfaction sub scale of 3 items likert scale. On other way almost 90% of the respondents were rated overall satisfaction scored above uncertain (neither agree nor disagree). Thus, 90% of the respondents satisfied with TB treatment service (Table 2).

**Table 2:** Overall patient satisfaction on TB treatment service at public health facilities of Sidama zone, South Ethiopia, from March to April 2011

**Items**	**Strongly disagree**	**Disagree**	**Neither agree nor disagree**	**Agree**	**Strongly agree**	
**I am totally satisfied with TB treatment service (N = 530)**	14(2.6%)	36(6.8%)	7(1.3%)	235(44.3%)	238(44.9%)	Mean 4.22
						SD 0.962
**Something about my consultation is better (N = 530)**	10(1.9%)	32(6.0%)	15(2.8%)	306(57.7%)	167(31.5%)	Mean 4.11
						SD 0.863
**I will advise my friends or relatives to see this provider. (N = 527)**	7(1.3%)	15(2.8%)	3(0.6%)	178(33.8%)	324(61.5%)	Mean 4.51
						SD 0.770
**The 10**^**th **^**percentile =10.**	Mean 12.79.					
**The 50**^**th **^**percentile =13**	SD ±2.30.					

### Adherence to medication

Using Morisky Adherence Scale the study revealed that the proportion of patient with poor adherence was 138 (26%). Of the 138 poor adherence 117 (84.78%) cited one or more reasons. The most commonly cited reasons for poor adherence were 58.97% absence of drug, 8.5% the health facility is far away from home, 6.8% too hard to take so many pills, 5.1% fear of interaction with other medication 5.1% had other appointment and 20.5% reported others which included felt depression, wanted to avoid side effect, busy with work (Table 3).

**Table 3:** Participant Medication Adherence Scale (MAS) items at public health facilities of Sidama zone, South Ethiopia, from March to April 2011, (N = 531)

**Medication Adherence Scale**	**Yes (1)**	**No (0)**
(MAS) score*		
**Forgot to take medication**	97(18.3%)	434(81.7%)
**Careless about taking medication**	24(4.5%)	507(95.5%)
**When feeling better, stopped taking medicine**	23(4.3%)	508(95.7%)
**When feeling worse, stopped taking medicine**	40(7.5%)	491(92.5%)

### General health status and treatment phase of the respondents as predictors of patient satisfaction

The health status of respondents and their treatment phase were significantly associated with patient satisfaction. The patient satisfaction score for good health status decreased by average of 0.653 (B 95% CI −1.044, -0.263) as compared to those who were in a very well health status. Similarly, the respondents who were not in good health status had an average decrease of 3.021 (B 95% CI −4.522, -1.519) on satisfaction score as compared to those who reported to be in a very well health status. Moreover, respondents on intensive phase of TB treatment had an increased satisfaction score by an average of 0.537 (B 95% CI 0.107, 0.9670) when compared to those who were on continuation phase (Table 4).

**Table 4:** Treatment aspect and Patients perception on health care provider interaction as predictor of patient satisfaction at public health facilities of Sidama zone, South Ethiopia, from March to April 2011

**Explanatory variable**	**No (%)**	**P-value**	**Un standardized B coefficient**	**Standardized B coefficient**	**95% CI for B**
Health status					
Very well *	288(54.2)				
Good	234(44.1)	0.001	−0.653	−0.141	−1.044, -0.263
Not good	9(1.7)	0.000	−3.021	−0.169	−4.522, -1.519
Treatment phase					
Intensive phase	155(29.2)	0.015	0.537	0.106	0.107, 0.967
Continuation phase*	376(70.8)				
Constant			1.919		0.856, 2.98
Perceived professional care		0.00	0.138	0.315	0.099, 0.178
Perceived time spent with health care provider		0.00	0.354	0.156	0.170, 0.538
Perceived accessibility		0.000	0.230	0.185	0.132, 0.328
Perceived technical competency		0.001	0.100	0.106	0.040, 0.160
Perceived convenience (cleanliness)		0.004	0.162	0.099	0.52, 0.272
Perceived consultation and relational empathy		0.007	0.029	0.109	0.008, 0.050
Constant			10.914		
Perceived waiting time		0.00	0.262	0.271	0.183, 0.342

*Reference category (highest frequency taken as reference category).

### Predictors of patient satisfaction with Patient perceptions on health care provider interaction and TB treatment service

Perceived professional care, perceived time spent with health care provider, perceived accessibility, perceived technical competency, perceived convenience and perceived consultation and relational empathy were identified as independent predictor of patient satisfaction on TB treatment service.

The respondents perception on professional care become increased by one unit, an average increase of 0.138 (B 95% CI 0.099, 0.178) on satisfaction Score. Likewise when the respondents perception on time spent with health care provider increased by one unit there was an average increase of 0.354 (B 95% CI 0.170, 0.538) on satisfaction score. In the same way, for one unit increase of the Perceived accessibility, the respondents had an average increase of 0.230 (B 95% CI 0.132, 0.328) on satisfaction score. The Perceived technical competency, Perceived convenience (cleanliness) and Perceived consultation and relational empathy had similar effect on the above explanatory variable. In addition to this the Perceived waiting time was significantly associated with patient satisfaction (Table 4).

**Table 5:** Treatment service aspect, Patient perceptions and satisfaction with health care provider interaction as predictor of adherence to TB treatment at public health facilities of Sidama zone, South Ethiopia, from March to April 2011

**Explanatory variable**	**P-value**	**B coefficient**	**OR**	**95.0% C.I. for OR**
Waiting time	.001	.022	1.022	1.009, 1.0035
Treatment phase	.000	−1.222	.295	0.172, 0.507
Constant	.000	−1.163	.313	
Perceived time spent with health care provider	0.00	−0.354	0.702	0.583, 0.845
Perceived accessibility	0.00	−0.225	0.798	0.721, 0.884
Perceived waiting time	0.011	−0.103	0.902	0.832, 0.977
Perceived professional care	0.006	−0.049	0.952	0.919, 0.986
Patient satisfaction	0.019	−0.095	0.90	0.840, 0.984
Constant	0.761	0.157	1.17	

### Health service waiting time and treatment phase as predictor of adherence to TB treatment

Patient’s treatment phase and waiting time in reception room was independent predictor of Patient adherence to TB treatment. The odds of poor adherence of the respondent was 0.295 (Adjusted OR 95% CI 0.172, 0.507) times higher on intensive phase as compared to continuation phase independent of waiting time. However, at one minute increased of patients waiting time at reception room, the poor adherence of the respondents was 1.022 (Adjusted OR 95% CI 1.009, 1.0035) times higher independent of patients treatment phase (Table 5).

### Predictors of adherence to TB treatment with Patients perception on health care provider interaction

Perceived time spent with health care provider, perceived accessibility, perceived waiting time and perceived professional care were identified as significantly associated with patient adherence to TB treatment. The odds of poor adherence of the respondent for one unit increase of patient Perceived time spent with health care provider was 0.702 (unadjusted OR 95% CI 0.583, 0.845) times higher. By one unit increase of patient Perceived accessibility, the odds of poor adherence of the respondent was 0.798 (unadjusted OR 95% CI 0.721, 0.884) times higher. Similarly for one unit increase of the Perceived waiting time, the odds of poor adherence of the respondents was 0.902 (unadjusted OR 95% CI 0.832, 0.977) times higher. The Perceived professional care had similar effect on adherence to treatment (Table 5).

### Patient satisfaction as a predictor of adherence to TB treatment

The overall Patient satisfaction had a significant association with adherence to TB treatment. This indicates that for one unit increase on the patient satisfaction score, the odds of poor adherence of the respondent was 0.90 (Unadjusted OR 95% CI 0.840, 0.984) times higher (Table 5).

## Discussion

This study attempted to investigate the level of patient satisfaction and adherence to TB treatment and associated factors among TB patients on intensive and continuation phase of treatment. The overall satisfaction rate with TB treatment service in this study is consistent with the study done India, where 91% of the patients in the study group expressed Satisfaction with the DOT services [20]. However, in this study those who are not coming to health facilities because of less satisfaction with the services were not included.

The study also showed that patients on intensive phase of treatment had an increased satisfaction score as compared to those who were in continuation phase. The reason for this might be patient on intensive phase has frequent and ongoing interaction with health care provider more than patients on continuation phase. Similar finding was reported by earlier study done on out patient satisfaction, where frequency of visit to health facility significantly associated with patient satisfaction [18].

Perceived professional care, perceived time spent with health care provider, perceived accessibility, perceived technical competency, perceived convenience (cleanliness) and perceived consultation and relational empathy were independent predictors of patient satisfaction (P < 0.05). This indicates a good patient provider relationship has a positive outcome on patient satisfaction. This finding is supported by the Study done in Tanzania, where good patient-service provider relationship was an important reason for satisfaction on TB treatment service [21] and previous finding on out patient satisfaction in study from Ethiopia [18]. In addition to this, perceived waiting time was significantly associated with patient satisfaction. This is consistent with study done in Eastern Ethiopia, where the level of satisfaction decreased with an increase in perceived length of waiting time [22].

The level of treatment adherence in this study is lower than the study conducted in South Africa where non adherence was reported to be 34% [23]. However our finding is higher than the 5.4% reported from Nigeria [24]. The reasons for poor adherence as reported by the respondents were absence of drug, the health facility being in far away from home, too hard to take many pills, fear of interaction with other medication, felt depression. Some of these reasons are similar to what was previously reported in earlier studies [25-27].

Patients’ treatment phase and waiting time in reception room were independent predictors of patient adherence to TB treatment. Patients on intensive phase, the respondents had good adherence to their TB treatment. This might be on intensive phase patients take their medication daily in the health institution and Patient might have good adherence during intensive phase, probably the illness is severe and fear of illness consequence make them not to miss pill. This is comparable with the study done in Hossana, Jimma and Arsi Zone of Ethiopia, where defaulting was highest during the continuation phase of treatment even if default not equal to poor adherence [16,25,26].

Our study demonstrated that perceived time spent with health care provider, perceived accessibility, perceived waiting time and perceived professional care had a positive association with patient adherence to TB treatment (P < 0.05). This is consistent with the studies conducted in South Africa and India [20,23]. The key challenge of direct observation of treatment is to implement it well, maximizing convenience and respectful interaction with patients [28]. Moreover, patient satisfaction was a significant association with adherence to TB treatment. An increased the overall patient satisfaction on TB treatment service has a positive effect on patient adherence to TB treatment. This is consistent with the study done in South Africa, where higher patient satisfaction with the service at the hospital was significantly associated with higher levels of adherence [23]. Similarly, study done in India stated dissatisfaction with services provided was the predictors of default [20].

## Conclusions

Majority of the respondents were satisfied with TB treatment service. However more than one in forth patients had poor adherence to TB treatment. Absence of drug, distance from treatment site, long waiting time at reception room were barriers to adherence.

Therefore, improving the treatment service process, maintaining close relationship between providers and patients, reducing waiting time in reception room will have a positive outcome on reducing poor adherence to TB treatment.

## Competing interests

We declare that we have no competing interests.

## Authors’ contributions

All the three authors were involved in the design, conduct of the study and the statistical analysis and interpretation of the findings. The authors read and approved the final content of the manuscript.

## Pre-publication history

The pre-publication history for this paper can be accessed here:

http://www.biomedcentral.com/1472-6963/13/110/prepub
